# Love and Home

**Published:** 1890-11

**Authors:** 


### LOVE AND HOME.

Sympathy and encouragement, love pats and hand clasps, light caresses and a warm, sheltering pair of arms, into which a girl may creep, are not listed in the programme of exercise in the school or home, but they can no more be dispensed with than air and sunlight. Love is a tonic, an overdose of which will never do any harm, and the child that gets a liberal allowance at home is not so likely to have her heart torn and her head turned by a first romance as the girl who goes through her teens heart-starved. Knowing how far a little loving goes, it is strange that mothers and elders should be so economical of it in dealing with their children.

Call your snub-nosed, freckle-faced Maria "sweet girl," and watch the beauty that will flash from her eyes. Put one arm about her scrawny little neck and draw her close to you if you would feel the love and gratitude of snuggling youth. You need not flatter her to stimulate her, but you can talk her into beauty, grace, elegance and worth. Sweet words are the music of the world. Say her hair is silky, tell her you like the smell of it and the touch of it against your face, lament your inability to arrange the front prettier, and you have made her stock of carroty wool a glorious halo and solved the hair question for life. Just so sure as she can lay her hands on a comb and brush, she will care for and dress her hair, and your face will come before her every time she looks in the glass. It may seem emphatic to tell your little sister that she is as round shouldered as a huckster, that she has hands like a hod carrier, that she walks like a duck and you think she must be web-footed. There are better, sweeter ways, however, to correct these faults. Suppose you send her home an engraving of a famous beauty or an artist's proof of some ideal woman ; try the effects of a shoulder cape, an inexpensive necklace or a chaplet in lace or ribbons, and as you put it about the small woman say in a whisper, "You will remember to stand straighter, won't you, dear ?" and kiss her before she has a chance to reply. A gentle woman in East Thirty-fourth street, who rules by love, has a young lady daughter of indescribable charm, although destitute of a single feature that could be called beautiful. About the walls of her room are pictures of queens, famous society beauties, singers, actresses and women that have been idolized by poets and painters. These prints have been the inspiration of the child and from them she has borrowed her grace of carriage, her "high little head" and the captivating manner that she makes the world accept for beauty.

If you want to cure a small boy of cowardice talk to him of heroes and heroism, aud if you want your daughter to make the most of herself and to be proud of you as a teacher and mother, keep the beautiful constantly before her. You know how you bought cosmetics to beautify yourself, how you followed the receipts for removing freckles and burnt all the skin off your face. You remember, too, the bottle of laudanum you kept in your trunk for murderous purposes, and the quantity of patent medicine you swallowed on the sly in the hope of getting fat, removing tan or becoming as fair as an Oriental lily. You could save the gullible little girl all this expense, misgiving, disappointment and physical derangement, by telling her how beautiful and fair she is, how glorious her eyes are and how more than priceless her youth is. Put her at her ease, get her *en rapport* with herself and you will make a beauty of her. Feed her heart and mind as well as her body. Tell her you love her, call her sweet names, reserving plain Maria for company. Get her confidence, cover up the blemishes by enlarging on her good points. Select ideals for her in marble, history, pictures, music and poetry, and though she may not reach any one of them the attempt will do her a world of good.

The daughter does not know you love her when you are eternally saying, "I am ashamed of the way you walk;" she cannot think she is dear to you when your observations are based on her defects, for which you are responsible. She does not know the cost of her living—and there is wisdom in her ignorance, by the way—nor the extent of your anxiety for her future welfare. She is unhappy. She wants to be loved and petted and cuddled and kissed, and if she does not find admirers, friends and lovers in her own family, she will find them in some other community. Of this be very certain ; your little girl is just what you make her. She is a divine study and worth all the care and attention, all the nurturing and devotion you can lavish on her.—*Truth.*

